# Letter from Augusta

**Published:** 1886-05

**Authors:** 

**Affiliations:** Augusta


### LETTER FROM AUGUSTA.

THE MEDICAL ASSOCIATION OF GEORGIA—THE PRESIDENT'S ADDRESS—A PRESIDENTS' PRIZE ESSAY FUND—PAPERS, NOTES, ETC.

*Editors Atlanta Medical and Surgical Journal :*

The thirty-seventh annual session of the Medical Association of Georgia was held in this city April 21, 22 and 23. There was a large attendance of physicians from all sections of the State.

Dr. Eugene Foster, the retiring President, in introducing the

President-elect, Dr. R. J. Nunn, of Savannah, thanked the Association for the honor conferred on him during the past term, and said that it was with great pleasure he announced the Association free from debt with a balance in the treasury. He attributed this desirable result to the efficiency of the Treasurer and Secretary Drs. E. C. Goodrich, of this city, and James A. Gray, of Atlanta.

The inaugural address, by Dr. Nunn, was an excellent paper, full of thought and well delivered. It was listened to attentively throughout. This address will, in the opinion of your correspondent, prove of more real value to the Association than any delivered before that body in a long time. It was eminently a practical address, suggesting many changes to the Association, which, if adopted, can but redound to the good of the Association. One evidence of this fact is the raising of a prize essay fund, at this meeting, of $600, which was the direct result of a recommendation, by President Nunn, that whenever a President is elected the trustees of the prize essay fund will expect to receive from him $100, to be placed to the credit of this fund.

The President-elect this year, Dr. T. O. Powell, of Milledgeville, cheerfully contributed this amount. So far, the only ex-Presidents who have contributed to this fund are Drs. R. J. Nunn, Eugene Foster, DeSaussure Ford, Henry F. Campbell and A. W. Calhoun. It is expected that every ex-President will contribute to this fund by the time of the next meeting. If so, then there will be to the credit of the Presidents' Prize Essay Fund about $1,500. Each year will add an extra hundred to the fund. It is safe to say that in ten years the Presidents' Prize Essay Fund, as suggested and put into practice by President Nunn, will reach very nearly the sum of $4,000.

This is only one of the valuable suggestions made by Dr. Nunn. There are many others, but space will not allow their mention now.

The different sections—Practice, Surgery and Gynecology—were well represented, most of them presenting valuable reports, which were read and referred to the Committee on Publication, and which will no doubt appear in the Transactions.

There was a larger number of voluntary papers submitted than is usual, and it is fair to say that the most of them possessed a higher order of merit than is common for such papers. Two of these papers which were read are entitled to special mention. The paper of Dr. J. P. Logan, of Atlanta, on the "Relation of the medical profession to alcoholic uses and abuses." No paper was probably ever read before the Association which was listened to with closer attention or elicited more favorable comment.

The paper read by Dr. H. McHatton, of Macon, on "Malarial Hœmoglobinuria," is a valuable contribution to the literature of the subject. The Doctor takes strong grounds against calomel in the treatment of this disease, and urges the free administration of quinine hypodermically.

Besides these papers there were quite a number of other voluntary papers. We mention those of Dr. R. O. Cotter, of Macon, " Certain syphilitic affections of the eye and ear."

Dr. George C. Hummel, of Savannah, "Pathology and treatment of gonorrhœa."

Dr. Eugene Foster, of Augusta, " A review of Antiseptic midwifery."

Dr. Thomas R. Wright reported and read a paper on " Surgical cases."

Dr. G. W. Mulligan, of Washington, Ga., " Stricture of the œsophagus in a child two years old."

Dr. W. H. Doughty, Jr., "Use of cocaine in certain operations."

Dr. Theodore Lamb, " Importance of the early recognition and treatment of plueritic effusions—the dangers of delay and routine methods."

Dr. Henry Gaither, Oxford, Ga., " Synopsis of country practice a half century ago."

Dr. A. G. Whitehead, of Waynesboro, "Is it only a coincidence?"

Dr. L. E. Borcheim, of Atlanta, "Causes and treatments of infants' diarrhœa."

Dr. Robert Battey, of Rome, Ga., "Antisepsis in ovariotomy and Battey's operation. Seventy consecutive cases with sixty-eight recoveries and two deaths."

Dr. R. J. Nunn, of Savannah, "Ramie—its use in gynecology."

Dr. V. H. Taliaferro, Atlanta "Tampons to the cavity of the uterus, etc."

Dr. A. W. Calhoun, Atlanta, "The other side of cocaine—the bad side."

The officers for the ensuing year are: President, Dr. T. O. Powell, Milledgeville; First Vice-President, Dr. G. W. Mulligan, Washington; Second Vice-President, Dr. E. H. Richardson, Cedartown; Treasurer, Dr. E. C. Goodrich, Augusta; Secretary, Dr. James A. Gray, Atlanta.

The next place of meeting is Atlanta, on the third Wednesday in April, 1887.

The following Committee of Arrangements was appointed by the President: Drs. J. F. Alexander, J. B. Baird, J. S. Todd, A. W. Calhoun, E. L. Connally, James A. Gray, W. P. Nicolson, W. S. Elkin, Virgil O. Hardon and J. C. Olmsted.

Augusta has long been noted for her eminent men in the medical profession. It was the home of the lamented Anthony, Ford, Dugas, Eve, and others whose names have been written on the roll, "In Memoriam." There are yet shining lights in the profession here; among them I mention the venerable Dr. Henry F. Campbell, the genial and jolly Foster, smiling and clever Goodrich, and a score of others which space will not allow me to mention.

Yours truly,

Augusta, April 23. CLIFFORD.

